# Who is providing pain care? Mapping chronic pain services across Scotland using freedom of information requests

**DOI:** 10.1177/20494637251413581

**Published:** 2026-01-07

**Authors:** Cassandra Macgregor, Christopher Seenan, Sivaramkumar Shanmugam, David N Blane

**Affiliations:** 1 Department of Physiotherapy, School of Health and Life Sciences, Glasgow Caledonian University; 2 Chronic Pain Service, NHS Lanarkshire; 3 General Practice and Primary Care, School of Health and Wellbeing, University of Glasgow

**Keywords:** chronic pain, pain care, interdisciplinary, freedom of information, health inequalities, health systems

## Abstract

**Background:**

The Scottish Service Model for Chronic Pain advocates multidisciplinary provision of care, stratified across sectors. However, there is lack of transparency over staffing capacities and provisions.

**Method:**

We submitted Freedom of Information requests to the 14 regional Scottish Health Boards in September 2024. We conducted descriptive quantitative and qualitative analysis of responses.

**Findings:**

We received 13 responses from 14 Health Boards with varying levels of detail. We found that staff groups most commonly reported to provide dedicated care for chronic pain are: medicine, physiotherapy, psychology and nursing, with lower levels of occupational therapy and pharmacy provision. Six Boards reported at least 1.0 whole-time equivalent medical, physiotherapy and psychology staffing capacity per 500,000 population, with sparse provisions by some populous Boards, showing considerable variation. A variety of pain groups were reported. Boards with higher levels of multidisciplinary staffing and group provision tended to report a dedicated management resource. We found several examples of cross-sectoral connections, potentially improving access to pain care according to need at the local level. The most variable and least well-developed responses were to the question on equitable delivery of care, demonstrating need to improve delivery of equitable approaches and strategies to pain care.

**Conclusions:**

We used FOI requests to collect data on chronic pain staffing capacity showing considerable variation across Scotland. There are limitations to this method; it is likely that our findings do not show a complete picture, rather useful insights into activities and provisions of services for pain care across Scotland.

## Background

Chronic pain is a heterogeneous and broad category of conditions and pain states where the pain has persisted for over 3 months, associated with significant disease burden and socioeconomic cost.^1–3^ Like many other health conditions, chronic pain is socially patterned, ranging from 29% prevalence in the least socioeconomically disadvantaged areas in Scotland, to 50% in the most,^4^ experienced more severely by a smaller number of people impacted by the pain emotionally, financially and in terms of daily function.^5–7^ Furthermore, there are higher levels of chronic pain among women and other groups marginalised by historic, economic and social conditions.^8,9^

Clinical guidelines for chronic pain recommend: that consultations are personalised and relevant bio-psycho-social factors assessed; provision of pain rehabilitative approaches including exercise, education, training in pain management skills, psychological approaches and pharmacological management.^10–13^

Most people in Scotland receive healthcare input for chronic pain through primary care.^4^ Smaller numbers attend chronic pain services through secondary care, where amongst other specialist services, outpatient Pain Management Programmes (PMPs) may be provided with a more intensive (usually residential) PMP available nationally.^14^ The Scottish Service Model for Chronic Pain was developed following recognition of chronic pain as a Long-term Condition (LTC) in 2008. The model advocated that regional Health Boards adopt a stratified, interdisciplinary model across levels of the health sector, including staff groups of: occupational therapy (OT), pharmacy, physiotherapy, nursing, medicine and psychology.^15^

The journey through healthcare for people with chronic pain is often non-linear, can prove stressful and challenging, with difficulties accessing pain care, information and receiving an appropriate diagnosis.^16–18^ Co-occurrence of multiple LTCs, distress, trauma and demands such as paid work or caring responsibilities may all impact coping with chronic pain, engaging with care and attending appointments.^17,19,20^ Many of these factors are subject to the negative impact of inequalities and more challenging for those already coping with the work of poverty and marginalisation.^19,21–23^ There are concerns over high levels of pain medication prescribing, particularly in areas of socioeconomic disadvantage.^24,25^ Health inequalities are widening in Scotland, with the health of those living in the most socioeconomically disadvantaged areas becoming more detached from the rest of the population.^26,27^

Delivering care to people with chronic pain requires availability of sufficient and appropriate staffing capacity, training and strategic coordination of care to meet the needs of the heterogeneous populations with chronic pain. Delivery of pain care involves working across service interfaces, community and leisure sectors. Service structures, and ways of delivering care and staffing, are not static phenomena, rather they evolve over time, cognisant of the need to reconcile research evidence with practical delivery and access issues. Movement of delivery is towards interdisciplinary care at local, community levels that is supported by specialist services for complex cases and training.^28,29^ In Scotland and the wider UK, this means increasing roles for multidisciplinary staff in primary care which requires working with the realities and restrictions of the GP contract.^30,31^ Furthermore, given the importance of activity and exercise options for people with chronic pain and other LTCs, connections and support through local groups and leisure services is important. Delivery of quality pain care – the aim of the Scottish Government^29^ – requires adequate staffing capacities, and embedded approaches to support pain care across sectors.

There remains limited publicly available data on how services are delivered across regional Health Boards in Scotland, including lack of transparency regarding the configuration, capacity and distribution of specialist staffing and service models for chronic pain. Understanding these variations is crucial to informing equitable and evidence-based policy responses. We aimed to obtain a current understanding of: staffing capacity dedicated to chronic pain care across Scotland, provision of pain groups, approaches to support generalist services and provide equitable care.

## Method

In Scotland, anyone can submit a Freedom of Information (FOI) request to a public service under the FOI (Scotland) Act 2002, within the remit of the legislation.^32^ FOI requests have been used since the inception of the legislation to obtain data not otherwise available about aspects of health services in the UK, with varying degrees of data successfully obtained.^33^ FOI cannot be used to obtain personal details, information that is already publicly available, or requests that are too costly to retrieve.^32^ FOI requests do not require ethical approval, and following FOI release, responses are placed in the public domain,^34^ often the Health Board website. Regardless of whether formal ethical approval is required or not, ethical considerations should be an ongoing concern throughout research, service evaluation and data collection including through FOI requests.^35^ We therefore approached the analysis constructively (seeking to ‘do good’ with research) and disseminated findings and recommendations to relevant and interested parties.

There is currently no specific reporting guideline applicable to FOI based data collection studies. However, we cover the five points suggested by Fowler et al. (2013) following their systematic review of FOI use in healthcare research,^33^ detailed in Supplement File 1. Furthermore, we prepared this paper with reference to general principles of transparency and rigour in health services evaluation. We selected the FOI approach over conventional survey or interview methods due to its feasibility, consistency and legal requirements for response. This enabled us to explore system level information not otherwise publicly available or routinely reported. No identifiable or patient level data were collected.

### Research setting

There are 14 regional Health Boards in Scotland; three small island Boards and 11 mainland Boards (although some of these Boards include island communities), ranging in size from Greater Glasgow and Clyde (GGC) with a population estimated at 1,192,000 people to Orkney with a population of 22,000.^36^ The Health Board populations vary in composition of geographies, nature of socioeconomic deprivation, ethnic backgrounds, access to services and transportation, and rural/urban settings. Population numbers of each Health Board are given in Table 2.

### Data collection

We submitted an enquiry under FOI legislation to all 14 regional Scottish Health Boards by email on 19 September 2024. The questions are shown in Table 1 with the full inquiry in Supplement File 2. We treated the responses as survey data subject to the nature of FOI.

**Table 1.** Questions submitted under FOI legislation to each health board.

**1) Staffing of services providing healthcare for chronic pain**
How much staffing capacity (WTE) does your health board provide for dedicated management of chronic pain (including secondary care chronic pain services, inpatient, or primary care clinics), for the following disciplines: Medicine, nursing, physiotherapy, psychology, pharmacy, occupational therapy? (As of 19 September 2024) What capacity does the chronic pain service manager have dedicated to chronic pain services (in WTE)?
**2) Provision of pain groups**
Please give details (including staffing and duration) of any groups for management of chronic pain that your board provides or funds, for patients. These groups may treat chronic pain as either as a stand-alone condition, or as part of general long-term condition management
**3) Chronic pain support for generalist services**
The recent Scottish health survey (2023) found that people with chronic pain mainly received healthcare support from their GP, and after that from a physiotherapist for chronic pain. What staff support/education does your board provide or fund for these staffing groups, for chronic pain management? What, if any, healthcare is provided directly by physiotherapists and GPs to patients for treatment of chronic pain?
**4) Equitable delivery of care**
Does your board have any strategies or policies, to address equitable delivery of care, providing resources according to level of need in more deprived areas?

### Data analysis

FOI responses depend on the knowledge and interpretation of staff responsible for replying, leading to potential variation in response quality and completeness. CM collated the responses as they were returned and checked for coherence. An apparent copy and paste error occurred in the Ayrshire and Arran (AA) response (there was a blank response and a potential answer to one question under the heading of a response to another question). CM queried with a contact there who provided the correct response as intended by the service lead. No other Boards were contacted to follow up details as all other responses appeared coherent.

CM collated the responses using separate Microsoft Word tables for each question (as per Table 1). Staffing numbers in numerical value were summarised in Table 2. A longer version with explanatory notes is in Supplement File 3. CM analysed the remainder of the responses using descriptive, qualitative analysis with minimal interpretation needed. The responses gave brief outlines of provisions in either numerical or text form. We were therefore limited in our interpretation of text or numbers to highly descriptive approaches. For example, we had insufficient text and details of context for thematic qualitative analysis.

**Table 2.** Staffing and group capacity of chronic pain services according to FOI responses.

Health board (population est. to nearest 1000)	Staff group total, in WTE, funded to provide chronic pain care (and group availability, as applicable)											
	OT	Pharmacy	Nursing	Medicine	Psychology	PT	CP service manager	PMP	Connected pain info session (one-off)	Other group	PAS	Exercise group
Highland (324,000)			2.2	0.7	2.0	0.7	0.8	y	y	y		
Grampian (587,000)			1.5	2.5	0.6	2.1	n/s	y	CAD	y		
Tayside (418,000)			7.21	3.79 OP + IP	1.6	1.8		y	y		y	
Fife (373,000)	1.04	1.9	4.77	1.1	2.3	6.32	1.0	y	y		y	y
Forth valley												
Lothian (919,000)			6.59 (A + C)	3.71 (A + C)	5.70	2.20	0.6 (OP service lead)	y	y	y		y
Lanarkshire (672,000)				1.2	1.0	1.5					y	
GGC (1,192,000)	0.5	0.2*	6.6	6.05	4.5	7.1	0.8	y	y	y		y
Ayrshire + Arran (366,000)	1	0.5	1	1.75	1.8	2.1	0.1	y	y		y	
Borders (117,000)	0.4		0.8	*		0.5			Y, PHD		y	y
D + G (146,000)				*	*	0.6					y	
Western Isles (26,000)											*y*	
Shetland (23,000)				*								
Orkney (22,000)												

Abbreviations and key: * = additional comment given in Supplementary File 3; OT = Occupational Therapist, PT = Physiotherapist, WTE = whole-time equivalent, CA = Consultant Anaesthetist, OP = out patient, IP = inpatient, CP = chronic pain, CPS = chronic pain service, GGC = Greater Glasgow and Clyde, D + G = Dumfries and Galloway, A + C = acute and chronic pain remit, CAD = community appointment day trial; PHD = pain hub drop in, y = yes, n/s = not stated, PMP = Pain Management Programme.

CM added the full responses for question topics two to four to Microsoft Word tables, with one file per question. She then summarised the responses according to the category or concept that was described in the response. For example, during analysis of the pain group responses, recurrent categories of PMP, and information session occurred in the data, and in the summary were labelled as such. Our findings are presented with: a narrative summary per question; Table 2; Tables 3 and 4 with collated summary points, with the responses in full in Supplement Files 3 to 6.

**Table 3.** Collated examples of support for generalist services.

• Ad hoc email contact.
• Meetings across services and staff groups to discuss care, complex cases, referrals.
• Shadowing chronic pain services.
• Structured in-service training.
• Tiered training and education programmes connected to tiered delivery of care.
• Pain champion network.
• Managed clinical network.
• National e-learning modules.
• Support to develop expanded role of multidisciplinary team (MDT) in primary care.

**Table 4.** Collated responses to equitable delivery of care question.

• Locate services in areas of socioeconomic deprivation, including new pilot projects
• Offer a variety of appointment modes including telephone, videocall and face to face, awareness of digital poverty
• Supported use of video calls to increase access in remote and rural areas
• Connections between local services and coordination of care
• Quality online materials to support care (NHS Lothian website)
• Recent work to address access for people who do not speak English or additional communication needs (GGC)
• Community link workers
• Advance nurse practitioner (ANP) to support patients with higher levels of need
• Seek guidance from local (health board) equality, diversity and human rights teams; public health; and equality impact assessment tools. Importance of local and community knowledge recognised
• Guidance from Scottish government pain management framework

### Reflexivity and positionality

CM is both a researcher, and physiotherapist at NHS Lanarkshire Chronic Pain Service, on a part time basis. SS and CS both hold academic positions only. DB works in both academia and as a GP in Glasgow. CM used insights from her clinical job in setting the question topics and wording. To minimise any potential influence of this positionality, or connections, on responses and therefore findings, CM did not inform any NHS colleagues of the FOI plans. CM was not asked to engage with the Lanarkshire response, and did not alert colleagues to the request. CM requested feedback from another clinical colleague and co-authors on her interpretation and allocation of categories in order to minimise any bias, however, the data analysis required minimal interpretation.

## Findings

We received responses from 13 of 14 Scottish Health Boards before 19 December 2024. Forth Valley noted they were unable to respond within the timescale. The level of detail within the responses varied. Some responses indicated that provision was through broader staff groups including acute and in-patient pain care and, in some cases, this meant the board could not provide a whole-time equivalent (WTE) figure. Additional notes on provision are in Supplement File 3.

### Dedicated staffing capacity for chronic pain management

Staffing capacity of Health Boards for provision of chronic pain care varied, with two island Boards reporting no dedicated staffing capacity for chronic pain. Three Boards – GGC, AA and Fife – employ staff across all six disciplinary groups surveyed. Notably, these three Boards also reported dedicated chronic pain service manager time. The most commonly employed staff groups for chronic pain reported by the Health Boards are: medicine (n = 11/13), physiotherapy (n = 10/13), psychology (n = 9/13), nursing (n = 8/13), with lower levels of OT (n = 4/13) and pharmacy (n = 3/13) staff. Six Health Boards employ at least 1.0 WTE medical, physiotherapy and psychology staff per 500,000 population, and these are Highland, Tayside, Fife, AA, GGC and Lothian. Lanarkshire and Grampian are comparable boards in size but do not employ this level of staff, or have dedicated OT, pharmacy or management. A summary of the information obtained from all Health Boards is in Table 2 with fuller details in Supplement File 3. A further point of note is that our data does not show the vacancy rate, rather staff posts that are funded.

### Provision of pain groups

The mainland Health Boards commonly offer a variety of pain groups accessed via different routes. Seven of the eight most populous, mainland Boards report that they offer a PMP, reported to range from eight to 13 weeks in length, and offer one session per week of between 2 hours and half a day. PMPs are by nature a multidisciplinary group programme that include assessment and recommended criteria for delivery.^13^ Responses are in Table 2 with further detail in Supplement File 4.

Provision of groups by Pain Association Scotland (PAS), a third sector organisation, was reported. PAS groups deliver educational material and self-management skills training.^37^ Five Boards, including three that do not offer any other pain group, reported that they provide PAS groups either online or in-person (Western Isles, Dumfries and Galloway, Tayside, Lanarkshire, AA). Furthermore, the PAS website reports that they offer groups in Fife and Borders.^37^

Six Boards reported that their services offer one-off pain education/information sessions, either aimed at patients in the community/primary care, or for patients referred to pain services. Borders additionally reported a pain hub drop in, and Grampian reported a trial community appointment day in the equity response (added here in case of a group element). Four Boards reported that they offered other types of groups including physiotherapy-led rehabilitative groups, a compassion therapy and a mindfulness group. Four Boards reported exercise-based groups and some of these were coordinated with leisure and local physiotherapy services.

### Provision of training and support for generalists

The questions in this section also covered provision of care and some responses were moved to the pain group section. Many responses acknowledged the vital role of generalist and primary care in delivering pain care, and that supporting these sectors was important. Responses from seven of the mainland Health Boards stated explicitly that their chronic pain services provided support to generalist services. Shetland and Orkney noted that their staff could access Grampian services for support and advice. Training and support took a variety of forms from ad hoc contact via email, meetings and shadowing chronic pain services to structured in-service training sessions. Examples of structured training and education programmes include the tiered approach for musculoskeletal (MSK) physiotherapy staff in Lothian; both the annual training programme and tailored sessions in AA; the Pain Champion network in Fife; and the managed clinical network operated in GGC. Three Boards noted access to recently developed online training modules for chronic pain management.^38^ Collated responses are shown in Table 3 with further detail in Supplement File 5.

The framing of the questions in this section gave physiotherapy and GPs as examples of generalist services meaning responses focused on these two staff groups. Four of the mainland Health Boards (Highland, Grampian, Tayside, GGC) noted the role of First Contact Physiotherapists (FCPs) in front line assessment and care for people with MSK chronic pain in particular. FCPs are trained to triage, assess, treat and refer people attending a local health centre or GP practice with a suspected MSK condition.^31^ Responses here acknowledge the need to support the FCP staff.

### Equitable delivery of care

Ten Boards gave a response to the question on equitable delivery of care, with another three stating that they did not hold the information and used an exemption available under FOI legislation. The responses to the equity question showed more variation and less well-developed approaches than the previous questions, suggesting scope for further development work in equitable delivery of care. There was awareness and acceptance of the need to address equity issues. The question gave the example of prevalence of chronic pain across deprivation areas – some Boards pointed to remote, rural and island geographies bringing particular access difficulties to provision of care (often associated with economic disadvantage) rather than distinct areas of socioeconomic disadvantage such as occur in GGC. With differing needs across Scotland, community/local knowledge is therefore clearly important. Connections between local and community services, and coordination of care also featured as a strategy. Collated responses are shown in Table 4, and full responses in Supplement File 6.

## Discussion

### Summary

The aims of this study were to determine capacities of NHS services for chronic pain across Scotland, including staffing numbers, provision of pain groups, and approaches to support generalist services and provide equitable care. Thirteen (of 14) Health Boards responded to the FOI request, returning varying levels of detail in their written responses. Medicine, psychology, physiotherapy and nursing staff are most commonly employed to deliver care for people with chronic pain. Few boards have dedicated pharmacy (n = 3/13) or OT (n = 4/13) provision for chronic pain. A variety of pain groups including PMPs and information sessions are delivered across Health Boards.

The FOI data provides evidence of considerable variation in staffing capacities and availability of group delivery across regions of Scotland. Boards with higher staff capacities, provision across multiple staff groups and those delivering a PMP tended to report dedicated manager time (often an HCP management resource). The leadership and coordination of the manager resource may therefore be an important feature of successful provision of pain care. There was recognition that chronic pain specialist staff play a role in supporting primary care, where most patients attend for pain care. The most variable, and least well-developed responses were to the question on equitable delivery of care. Given the pressing issue of health inequalities in Scotland, and that healthcare plays a role in this,^27^ there is scope to improve delivery of equitable approaches and strategies to pain care.

### Strengths and limitations

A strength of using FOI inquiry as a method to facilitate data collection can be that responses are returned, and in a standard way, although others have received fewer responses than us.^33^ The limitations of administering a service-level survey via FOI include gathering a snapshot at one point in time, and any potential uncertainty over the meaning and interpretation of questions. Furthermore, the knowledge and remit of the responder/s will influence what is included in the response. For example, although primary care clinics and third sector groups were included in the remit of the question, secondary care or administrative staff may not know details of wider provisions. While we consider dedicated management time as having a positive relationship with availability of care for chronic pain, it may also be that the services with dedicated management time were well positioned to respond to the FOI request. Collecting data in other ways could add to the picture. Another factor was that Boards did not need to provide the level of detail that some of them actually did, in particular, the detail on group availability. The implications of collecting the data this way are that we cannot claim to convey a full picture, rather useful insights into the nature of activities of services for chronic pain in Scotland.

The questions were framed in a particular way, influencing the details given in the responses, for example, those for generalist services are focused on medical staff and physiotherapists. The FOI exercise shows a limited frame of pain care delivery based on secondary care provision and responses, whereas most people with chronic pain attend primary care. The FOI exercise does not reflect the full variety of provision across sectors, and we do not show vacancy rates in our data. Our data provides insights into capacities for pain care, important to understanding structural healthcare provisions and inequalities. Our data does not show the full patient journey across sectors including inpatient, primary and community resources; what care is actually being provided by staff and whether this aligns with evidence-based recommendations, or any service outcomes. Our analysis does not account for the multiple factors that influence health needs of the population and affects funding settlements across Health Boards.^39^ Furthermore, we did not state whether the services were for adults, paediatrics or both and so this distinction cannot be drawn from the data, although the Faculty of Pain Medicine collected data on this recently.^40^

### Comparisons with literature and earlier Scottish data

Mellor^14^ published data on chronic pain services in Scotland in 2018 that included multidisciplinary staffing provision. She did not report WTE staffing numbers but did collect data on what staff groups were employed by Health Boards. Comparison of our FOI data shows similar distribution of staffing groups to 2018, with the number of Boards reporting specialist OT having increased from two to four. Delivery of PMPs through secondary care services has reduced slightly since Mellor’s report,^14^ and there may be a tension between full recommended delivery length and accessibility or capacity issues. The provision of pain groups is a broad picture and includes a more intensive PMP delivered nationally, and locally provided pain education/information sessions, and supervised exercise groups.

Exercise participation is an important health behaviour and recommendation; however, pain, lack of finances, and demands on time can be barriers to participation.^19^ Some people with chronic pain benefit from multimodal input (including the application of exercise and movement that is part of a PMP), tailoring of exercise approaches and support to uptake exercise.^13,41^ If pain services are working with individuals to overcome barriers to exercise participation, delivering supported exercise sessions, or connecting to leisure services to improve uptake and maintenance of exercise, this is to be encouraged. Understanding what does and does not work could be a valuable focus of evaluation.

We argue that the FOI findings show considerable variation of dedicated care for chronic pain across Scottish Health Boards with sparse provision in some areas, raising questions about whether quality pain care will be delivered on the ground, and who will deliver it. Reducing ‘unwarranted variation’ is an aim of Realistic Medicine (strategy of Scottish Chief Medical Officer) and is important to consider in the context of the *necessary* variation that occurs across Scottish regional Health Boards.^42^ The island health boards, for example, may not be expected to deliver a PMP, or have full specialist MDT staffing, rather to have a variety of support and access strategies for their residents and staff. The lack of staffing capacities and provisions of larger mainland Boards of Lanarkshire and Grampian could be framed as ‘unwarranted’; however, our analysis may not capture the full extent of their services.

The Faculty of Pain Medicine (FPM) conducted a Gap Analysis based on survey data of its membership,^40^ with a breakdown of responses across the four UK nations in the longer report.^43^ The 31 survey questions were based on the FPM 21 core standards for pain services,^44^ with framing towards medical staffing and services, for example, training of anaesthetic staff and provision of neuromodulation services. The FPM report concludes that there is need to improve access to: paediatric and cancer pain services; psychology; PMPs; research and development; and outcome data management support.^40^ The focus and questions were mostly different to ours, with some overlapping queries about staff and PMP availability, and as such both studies complement each other.

Recent work in Sweden focused on the creation of pathways for chronic pain across health sectors, similar to those for other diseases.^45^ The pathways connect primary to specialist care with the aim of delivering earlier interdisciplinary pain rehabilitation in primary care and reducing the current wide variation in pain care availability and practices. The Swedish working group recognise the need for initial investment in training and development to improve staff knowledge and skills on chronic pain and management, and to reduce unwarranted medical procedures and diagnostic tests.^45^ Process outcomes of the pathway are: percentage of patients diagnosed with chronic pain who have a rehabilitation plan in place within 1 month of diagnosis, and proportion of patients diagnosed with chronic pain who have a regular primary care contact 1 month after diagnosis.^45^ Areas for improvement in Scotland are access to quality care, receiving early information and a suitable ‘diagnosis’, and care from staff who are well informed about chronic pain.^17^ The Swedish approach to practical delivery could therefore be useful to follow.

### Addressing equitable delivery of care

The main point of uncertainty and variation in FOI responses were on strategies to address equitable delivery of care. The issues of local knowledge and tailoring equitable delivery to the needs of communities were raised by some responses. Community engagement and determining the specific needs of local populations is a significant feature of literature on mitigating inequities in delivery of care.^46–48^ There is evidence of some pain services involved in delivery of community exercise classes, community appointment days, and pain cafes; strategies that may improve access and prove equitable in the long-term, and could lend themselves to community engagement work. Potential approaches could further include locating services in, or close to community centres, including libraries and cultural/leisure centres, or providing targeted home visits.^46,48^

Improving connections between services and coordination of care also featured as strategies in some FOI responses, for example, community link workers in Orkney, and an ANP employed to support more vulnerable patients in Shetland. Employment of community link workers (and also welfare advice workers) is a strategy to improve health equity across Scotland.^30^ Interfaces between care sectors are recognised as a potential source of inequities; connections are therefore relevant to equity.^49^ It is clear that many services foster connection between primary and secondary care sectors, and community resources, for example, leisure, but not all. Services could strategically embrace connections with an equity lens, by further fostering equitable approaches and collecting adequate data to inform service development.

The most common response to the equity question was to offer a variety of appointment methods, using video call to potentially increase access, with awareness of digital poverty sometimes reported. Changes to healthcare delivery in response to the COVID-19 pandemic included greater use of online resources, telephone and video calls. Post pandemic, there are calls to carefully consider how digital healthcare is implemented so that its use does not exacerbate health inequalities, maintaining choice of appointments, avoiding digital-first approaches, improving access and enablers to use of digital resources at the societal level.^50,51^

The FOI question gave the example of differences in chronic pain prevalence across areas of high/low socioeconomic deprivation, potentially prompting responses towards ‘proportionate universalism’ – that services should be delivered universally but with a scale and intensity that is targeted towards disadvantage, that is, proportionate to need.^52^ Examples from some FOI responses were given as deliberate location of services and outreach hubs in areas of socioeconomic deprivation.

An important part of any strategy to address health and healthcare inequities is staff awareness and education on mechanisms of inequities and ways of addressing these. Recent literature syntheses of the general practice and musculoskeletal fields detail evidence-based strategies that could be utilised in pain care and to raise awareness of health inequities.^46–48,49^

### Future research and evaluation

Our data shows a combination of potential approaches to delivering pain care, supporting generalist staff and addressing equity, and a strength may lie in combining the most effective elements across Scotland in future work. However, a limitation of our work was in mapping chronic pain provisions across primary care and other sectors: future work could map these services across primary care, community and the third sector. Furthermore, collecting sufficient data including demographics is key to understand both chronic pain and equity outcomes from services and projects, and to prevent Intervention Generated Inequalities (IGIs).^46^ Understanding the context around health interventions is important, including other operations of the health sector, wider funding, policy and political decisions.^53^ Involving whole practice/service staff groups including administrative staff in redesign approaches are important to successful implementation of equitable strategies.^47,54^

Health outcomes are worsening for the 15% of Scottish population living in the most socioeconomically disadvantaged areas, and evidence of this is shown through hospital admissions, early deaths, medication prescribing levels and misuse, MLTCs, and complex social situations.^26,27,55^ Equitable responses are therefore needed across sectors from the acute/hospital sector to community resources. The FOI data showed that some pain services connect across outpatient and inpatient/acute sectors which could prove beneficial in any efforts to improve management for individuals marginalised by social circumstances, if strategically coordinated. However, there are limits to the capacity of the health service to mitigate health inequalities; therefore, broader policy change is an important target of any advocacy work.

## Conclusions

We used FOI requests to investigate chronic pain staffing capacities across Scotland and received 13/14 responses of varying levels of detail from regional Health Boards. We cannot claim to show a full picture of provisions for chronic pain due to the limited nature of our FOI snapshot, rather we provide useful insights into the situation. We found that staff groups most commonly employed to provide dedicated care for chronic pain are: medicine, physiotherapy, psychology and nursing. Six Boards reported at least 1.0 WTE medical, physiotherapy and psychology staff per 500,000 population, with sparse provisions by some populous Boards. A variety of pain groups were reported. The few Boards with staffing across the six disciplines surveyed, and PMP provision, also tended to report a dedicated management resource. We found several examples of chronic pain services across Scotland that have cross-sectoral connections, potentially improving access to pain care according to need at the local level. However, responses to equitable provision of care were variable, showing potential need to improve strategies here. Future research and development work could focus on analysis of system-wide approaches that disproportionately benefit marginalised groups, and integrate findings from wider literature fields. However, a prerequisite to any approach that can deliver quality pain care is sufficiently resourced services, particularly in areas of higher need.

## Supplemental Material

Supplemental Material - Who is providing pain care? Mapping chronic pain services across Scotland using freedom of information requests Supplemental Material for Who is providing pain care? Mapping chronic pain services across Scotland using freedom of information requests by Cassandra Macgregor, Christopher Seenan, Sivaramkumar Shanmugam and David N Blane in British Journal of Pain.
