# Supplementary material for: Who is providing pain care? Mapping chronic pain services across Scotland using freedom of information requests
Source: Br J Pain. 2026 Jan 7:20494637251413581. Online ahead of print. doi: 10.1177/20494637251413581 (PMC12779529; doi:10.1177/20494637251413581)
Supplement: Supplemental Material - Who is providing pain care? Mapping chronic pain services across Scotland using freedom of information requests [file sj-pdf-1-bjp-10.1177_20494637251413581.pdf]

Supplementary files

Supplementary File 1

Point 1: Which body/bodies were data requested from?<br>Detail under section 2.1, 2.2 and details of the Health Boards named in Table 1.
Point 2: How many and what questions were asked?<br>The questions are given in Box 1 and supplementary file 2.
Point 3: The rate and timeframe of responses.<br>Given under section 3.0 and each section in the findings
Point 4: Reasons given when data not released.<br>In section 3.0 – 13 responses given, 1 Health Board noting they were unable to respond within time frame.<br>Section 3.4 10 responses given to that question, with 3 boards noting they did not hold the information.
Point 5: Amount of data received

Supplementary File 2: Questions submitted under FOI legislation to each health board

**Staffing of services providing healthcare for chronic pain**
How much staffing capacity (WTE) does your health board provide for dedicated management of chronic pain (including secondary care chronic pain services, in-patient or primary care clinics), for the following disciplines: medicine, nursing, physiotherapy, psychology, pharmacy, occupational therapy? (as of 19th September 2024)<br>What is your vacancy rate for each of these capacities as of 19th September 2024?<br>What capacity does the chronic pain service manager have dedicated to chronic pain services (in WTE)?
**Provision of Pain Groups**
Please give details (including staffing and duration) of any groups for management of chronic pain that your board provides or funds, for patients. These groups may treat chronic pain as either as a stand alone condition, or as part of general long-term condition management.
**Chronic pain support for generalist services**
The recent Scottish health survey (2023) found that people with chronic pain mainly received healthcare support from their GP, and after that from a physiotherapist for chronic pain. What staff support/ education does your board provide or fund for these staffing groups, for chronic pain management?<br>What, if any, healthcare is provided directly by physiotherapists and GPs to patients for treatment of chronic pain?
**Equitable delivery of care**
The Scottish health survey (2023) found that chronic pain is a condition that is experienced unequally, with 29% prevalence in the least deprived decile in Scotland, ranging to 50% prevalence in the most deprived decile. Does your board have any strategies or policies, to address equitable delivery of care, providing resources according to level of need in more deprived areas?

Supplementary File 3: staffing capacity of chronic pain services according to FOI responses

**Health Board** (population est. to nearest 1000)	**Staff group total across all Bands given in WTE (as of 19/9/24)**							
	OT	pharmacy	nursing	medicine	Psychology	PT	CP service manager	Other/ notes
Highland (324,000)	0	0	2.2	0.7	2.0	0.7	0.8	Nurse - 0.9 WTE Clinical Manager, 1.3 WTE Senior Specialists<br>Psychology - 1 WTE Clin psych, 1 WTE CBT therapist
Grampian (587,000)	0	0	1.5	2.5	0.6	2.1	n/s	Medicine - 4 x Pain Consultants (3 PT, 1 FT)
Tayside (418,000)	0	0	7.21	3.79 OP + IP	1.6	1.8	no	Nursing - B8a WTE 0.81, B7 WTE 4.6, B6 WTE 1.8; Medicine - 1.75 WTE OP + 0.75 WTE IP (plus vacancies); Psychology - 0.6WTE B8b, 1.0WTE B8a (vacancy); PT - 1WTA B8 / 0.6WTE B7/ 0.2 rolling B6
Fife (373,000)	1.04	1.9	4.77	1.1	2.3	6.32	1.0	PT, OT, nursing all note assistants as part of WTE staffing group<br>Minor vacancies
Forth Valley								*No response*
Lothian (919,000)	0	0	6.59 (A+C)	3.71 (A+C)	5.70	2.20	0.6 (OP service lead)	Lothian operates 2 separate pain services; Medicine and nursing staff work in hospital, do not see OPs; Different managers contribute time to IP hospital service management.
Lanarkshire (672,000)	0	0	0	1.2	1.0	1.5	no	Medicine – '12 consultant sessions'<br>Manager – oversees 8 services – adjusts time depending on need
GGC (1,192,000)	0.5	0.2*	6.6	6.05	4.5	7.1	0.8	Pharmacy – 0.2WTE Pain Service and 0.4WTE Primary care, not funded by pain service, Vacancies noted
Ayrshire + Arran (366,000)	1	0.5	1	1.75	1.8	2.1	0.1	Mental Health Services: 1.5WTE dedicated pain psychology resource<br>Manager – 1 x session/ w or as needed, CPS has several senior clinical team leads.
Borders (117,000)	0.4	0	0.8	*	0	0.5	no	Manager - falls within the remit of the Clinical Services Manager – Planned Care role (1 WTE). Medicine capacity given as '6.57 PAs/ week'.
D + G (146,000)	0	0	0	*	*	0.6	no	2 x CA provide OP clinics - part of wider service - not possible to provide WTE. Psychology - part of clinical health psychology.
Western Isles (26,000)								*No dedicated services - access to generalist OT, PT, Pharmacy, Psychology,*
Shetland (23,000)				*				Employ generalists, no specific primary care clinics. CA with special interest in CP, provide OP clinics - not possible to provide WTE as part of wider service
Orkney (22,000)								*No dedicated service - part of overall Nurse, Consultant, PT roles. Patients are also seen within the NHS Grampian specialist service.*

Abbreviations and key: * = additional comment given in 'other/notes' column; OT = Occupational Therapist, PT = Physiotherapist, WTE = whole time equivalent, CA = Consultant Anaesthetist, OP = out patient, IP = inpatient, CP = chronic pain, CPS = chronic pain service, GGC = Greater Glasgow and Clyde, D+G = Dumfries and Galloway, A+C = acute and chronic pain remit

Supplementary File 4: Pain group provision – summarised responses per board

Summary - Provision of Pain Groups<br>Abbreviations: HCP = Health care Professional; MDT = Multidisciplinary team; PAS = Pain Association Scotland groups (not HCP staffing); PMP = Pain Management Programme (MDT, HCP staffing); ACT = Acceptance and Commitment Therapy; F2F = in person delivery, w = week; h = hour; v = virtual (online); CBT = cognitive behavioural therapy; PT = physiotherapy; in-p = in-person, CP = chronic pain, Ax = assessment	
Mainland Health Boards (ordered North to South, geographically)	
Highland	PMP v – 10w x 2h (2 groups running parallel using the ACT model combined with pain education and physiotherapy pain management).<br>Mindfulness Group v – 8w<br>Pain Education Group v – 1h, 2/month; intro for all recently referred patients.
Grampian	PT led ACTive with Pain group. 8w x 2.5h with additional content covered digitally and a 2.5h follow up session after 3 months (both v and F2F).<br>12w x 2.5h PMP and additional v content.
Tayside	PAS, PMP, and group patient education classes.
Fife	PMP 8w x 2.5h F2F. (incl. information session -1h v and individual Ax with psychology and PT pre-group. Delivered in geographical locations appropriate for w/l). Community Education sessions – for patients and carers (self-booking).<br>Due to pilot- MSK/Pain gentle exercise group, staffed by 2 PTs and 2 assistants (staffed between MSK, Pain and Fife Leisure), 12w x 1h program.
Forth Valley	No response 4/1/25
Lothian	PMP 10w x 3h (1 x pain education group offered prior to participation and individual Ax. Staffing = 2.2 WTE PTs and 4.7 WTE psychologists).<br>Pain Mx group- chronic pelvic pain - 8w x 2.5 -3h (staffed by 0.2WTE psychology).<br>Groups run by MSK PT team (some specific HSCP, not avail throughout): PT led group – 6w x 2.5h; Hydrotherapy Circuit classes- 6w; 'Escape pain' groups- 6w, x2 per week, 1h.<br>Edinburgh leisure groups- referral- Edinburgh HSCP
Lanarkshire	PAS
GGC	PMP (10 and 12 w groups, half day per w), 3 or 4 groups run per w.<br>Pain Early Information Sessions 1.5h – introduction to pain service.<br>Compassion Focused Therapy Group – approx. 4 per year.<br>Supported Gym Session Groups 1h – 3 groups per w, supported by PT<br>Intro to CP Mx – primary care 1-1.5h, approx. 2/ month; staff 1 x HCP, 1 x PMP graduate<br>Scottish National PMP (NSD funded, hosted in GGC) F2F = 3w (full days); v = 5w (half days).
Ayrshire + Arran (+ email query)	PMP – 13w<br>PAS groups – 1 x v, 1 x in-p each month<br>Pain Early Information Session - 1 x v, 1 x in-p each month
Borders	Pain education x 1h<br>Supported exercise classes 2 x 1h<br>Pain hub drop in 1hr
D + G	PAS
Island Health Boards	
Eileanan Siar Western Isles	PAS
Shetland	No pain groups. Voluntary Action Shetland has a community directory of support groups.
Orkney	No pain groups

Supplementary File 5: Chronic pain support for generalist services and provision of pain care

Chronic pain support for generalist services and provision of pain care<br>Note question focus on Scottish health survey reports pts with chronic pain mainly supported through GP and PT.<br>Abbreviations: CPS = secondary care chronic pain/ pain management service; PC = primary care; FCP = First Contact Physiotherapist; CP = chronic pain, PT = physiotherapist; Scottish Government Pain Management Framework = SGPMF; GP = General Practitioner (medic); MDT = multidisciplinary; PM = Pain Management; OT = Occupational Therapists; Mx = management; APP = Advanced Practice Physiotherapist; Ax = assessment	
Mainland Health Boards	
Highland	CPS supports PC through consultation incl. clinical dialogue.<br>FCPs provided with training and support from CP Lead PT.<br>Note SGPMF training and education materials for all staff available soon.<br>First line treatment for CP is carried out by GPs who can access a wider MDT within PC.
Grampian	Skilled practitioner course of 1.5 days to PC practitioners, delivered by CPS staff (accessed by Podiatrists, Nurses, PTs, OTs). GPs attend CPS monthly MDT meetings to discuss cases.<br>Note SGPMF training and education materials for all staff available soon.<br>FCPs and GPs, along with MSK and community PT teams support people with CP.
Tayside	MDT approach - GPs and PTs play central roles, particularly with MSK pain.<br>GPs - often first point of contact, role includes: initial Ax and diagnosis, pharma mx; referral for investigations or specialist care, long-term monitoring and care planning.<br>PTs, particularly FCPs, play a pivotal role in PC for patients with chronic MSK pain, their role includes: initial Ax and diagnosis, personalised programmes (tailored exercise, education, self-management and manual therapy), pain Mx strategies, referral to other services.
Fife	Support available: Protected Learning Time Presentations and workshops; guidelines for primary and secondary care; staff can shadow services delivered by CPS; Pain Champion Network; GPwSI training for GP surgeries; training directly offered to MSK PTs, pharmacy, GPs, Mental Health, nurses; Learn Pro module on CP; developing short videos for use of medicines; quarterly data packs to help GP practices reflect on own prescribing of pain medicines; annual analgesic report for prescribing in primary care.<br>Healthcare is provided directly by PTs and GPs to patients.
Forth Valley	Not received 4/1/25
Lothian	All MSK PTs receive training in CP Mx - level and extent of training is dependent upon role. MSK PTs provide care across patient pathway incl. in PC as part of MDT around the GP, OP services, primary/community and acute sector locations and interfaces between services.<br>PT education for pain Mx:<br>Level 1 - in-service training, and problem solving/ clinical reasoning session(s)<br>Level 2 - additional level two training (including completion of reflections)<br>Level 3 – additional - facilitate a level 2 training; development support from level 3 PT; attend triage sessions, MDT CPS meeting; shadow CPS group and individual sessions, reasoning and reflection sheets.<br>Provision of care and training of MSK staff supported by 2.2 WTE PTs from CPS.<br>Care provided individually or group, patients may be escalated depending on their needs.
Lanarkshire	Specialised PT and Psychology available through CPS.<br>PTs provide PT for the Mx of CP. GPs provide prescriptions for medication for Mx of CP.
GGC	MSK service is not dedicated to CP Mx, but does see patients with CP as part of remit (MSK service = 30 WTE APPs within GP practice, and main service = approx. 140 WTE staff - vacancy rates approx. 12-18 WTE).<br>APPs in PC act as first point of contact for 44% of population for MSK conditions.<br>APP and MSK service have regular contact with patients with CP and LTCs. Training – undergrad, ongoing CPD, internal training and funded external courses.<br>Managed Clinical Network Education Group across primary and secondary care – explore, support and deliver educational session for PC and GP practices; rolling educational programme for AHPs; all CPS HCPs involved in ad hoc requests for training.<br>'Chronic Pain Management in Primary Care' Guidelines: https://rightdecisions.scot.nhs.uk/ggc-referral-management/pain-management/?organization=nhs-ggc

Ayrshire + Arran	The CP PT offers all MSK PTs training. CPS offers PC information sessions and 1-1 support. The MDT CPS provides annual Education days and specific PC events, incl. bespoke training packages to GP trainees, GP practices, AHPs, Prison Service, Mental Health services. Pilot work within large GP practice to improve communication and skills.
Borders	CPS provides: email advice, peer support teaching as requested, close links with PC CP Pharmacist, access to Pain Hub for PC patients, and third sector organisations.
Dumfries + Galloway	Staff identify learning needs and may access courses on CP Mx. NHS D+G cannot comment regarding GPs, as they are independent practitioners - source and access their own training needs. Notes provision by CPS and that GP and PTs provide care for CP as a part of daily work.
Island Health Boards	
Western Isles	Ad hoc support from PAS, incl. staff can shadow sessions.
Shetland	Generalists can access decision making support via the Consultant Anaesthetists or NHS Grampian CPS as needed. Patients are currently seen within the MSK pathway for PT by MSK generalists.
Orkney	Staff support from NHS Grampian CPS if required. Patients will be seen and treated by PTs and GPs on the basis of individual needs.

Supplementary File 6: Equitable delivery of care

Equitable delivery of care	
Mainland Health Boards	
Highland Na Gaidhealtachd	The Chronic Pain Management Service is a small service working (5.6 WTE clinicians) with patients referred from their GP across NHS Highland, including Argyll and Bute, through face to face, telephone and NEARME appointments. Following first line treatment in primary care, patients are vetted on referral to the Chronic Pain Team by the Multidisciplinary Team (Nursing / Psychology / Physiotherapy) for appropriateness of referral and urgency. Patients are then seen on date of referral order, regardless of their geography, unless they are identified as requiring a more urgent appointment – i.e.: Chronic Regional Pain Syndrome. Whilst we do not have access to data around our patient's deprivation levels, we do our best to overcome accessibility difficulties related to our remote and rural geography, often associated with economic deprivation, through delivery of services using virtual or telephone means where possible.
Grampian	NHS Grampian are running a test of change – offering Community appointments days to people living with chronic pain. The first test site for this offer is being intentionally delivered in an area of higher deprivation within Grampian to attempt to address this inequality. Further information on this test of change can be found at the following links: More than 120 patients seen at NHS Grampian event (https://www.nhsgrampian.org/news/2024/september/more-than-120-patients-seen-at-nhs-grampian-event/) https://www.youtube.com/watch?v=O20YH8vXAUA
Tayside	NHS Tayside has no specific strategies or policies pertaining to chronic pain in this regard. Exemptions Section – Application of Freedom of Information (Scotland) Act 2002 exemptions and Data Protection Act 2018 Principles.
Fife	Any changes to service are considered under EQIA and mitigations put in place where possible.
Forth Valley	No response received as yet.
Lothian	In order to address some of the difficulties in accessing care in this population, pain management services are now provided by telephone, online videocall or face to face, according to patient preference. Pain Management and Pain Education Groups are delivered both online and face to face. We are aware of the issue of digital poverty and will always try and facilitate patient access wherever possible. Outreach models such as pain management projects in the community as a way of increasing access have been considered but are not yet resourced. The NHS Pain Management Service has developed a website which contains information on a range of self-help

	materials including videos and links to other pain management resources and support, including third sector services.
Lanarkshire	NHS Lanarkshire does not collect the data referred to. Therefore we do not hold strategies or policies to address equitable delivery of care in this particular area. Accordingly, I must advise that in terms of Section 17 of the Freedom of Information (Scotland) Act 2002 the information sought is not held.
GGC	The Secondary Care Chronic Pain Service offers person centred models of care, recognising that many individuals with chronic pain come from the lowest Scottish Index of Multiple Deprivation (SIMD) rankings and thus can be affected by many of the wider determinants of health. The service is fully engaged with the Service Delivery Framework: Implementation Plan (2022) with a number of clinical staff from across the service being involved in short like working groups.<br>All parts of the pain service offer patient choice for accessing the service between face to face and virtual options – thus reducing inequalities for potential digital exclusion. Significant work has also been undertaken addressing access to the service for patients who do not speak English or who have additional communication needs – this has been in conjunction with NHSGGC Equality and Human Rights team.
Ayrshire + Arran	The Pain Services work to nationally agreed standards, “Framework for pain management service delivery” and in partnership with Public Health and Equality and Diversity colleagues.
Borders	The hospital and most facilities local to the most deprived areas in the region currently provide outreach pain hubs where possible - in South and East of the region which are the second and third most deprived areas. NHS Borders also has good links with Live Borders.
Dumfries + Galloway	NHS Dumfries and Galloway do not hold this information; therefore, this request is refused under section 17 of FOISA.
Island Health Boards	
Eileanan Siar Western Isles	Through development, the Pain Association sessions have all moved online although there remains the option of 1:1 if need be. In the past this has been found to have little demand and more individuals are happy to attend online. This provides more equitable access, and the local IT department or practices can facilitate set up if patients require.
Shetland	Shetland is a rural island community and does not have distinct areas of deprivation. Deprivation is experienced by individuals and families who live across the isles and it is difficult to take a targeted approach, but we have joined up working between primary, secondary, community and social care – with GPs as care co-ordinators. There is an ANP employed to help support people who are more vulnerable and may need additional support. We also have living well hubs in our communities to bring services together to reduce inequity of access.
Orkney	Orkney is a small rural island community and does not have distinct areas of deprivation. Pockets of deprivation are experienced by individuals and families who live across the isles, the biggest gaps are seen in our ferry-linked isles and most remote parishes.<br>Community Link Practitioners can help arrange access to services, NHS Orkney has increased the use of virtual appointments which enables patients experiencing access deprivation to receive equitable services to other members of the community.
